# Isoelectric point-based fractionation by HiRIEF coupled to LC-MS allows for in-depth quantitative analysis of the phosphoproteome

**DOI:** 10.1038/s41598-017-04798-z

**Published:** 2017-07-03

**Authors:** Elena Panizza, Rui M. M. Branca, Peter Oliviusson, Lukas M. Orre, Janne Lehtiö

**Affiliations:** 1 Department of Oncology-Pathology, Science for Life Laboratory, Karolinska Institutet, Stockholm, Sweden; 2 GE Healthcare Bio-Sciences AB, Uppsala, Sweden

## Abstract

Protein phosphorylation is involved in the regulation of most eukaryotic cells functions and mass spectrometry-based analysis has made major contributions to our understanding of this regulation. However, low abundance of phosphorylated species presents a major challenge in achieving comprehensive phosphoproteome coverage and robust quantification. In this study, we developed a workflow employing titanium dioxide phospho-enrichment coupled with isobaric labeling by Tandem Mass Tags (TMT) and high-resolution isoelectric focusing (HiRIEF) fractionation to perform in-depth quantitative phosphoproteomics starting with a low sample quantity. To benchmark the workflow, we analyzed HeLa cells upon pervanadate treatment or cell cycle arrest in mitosis. Analyzing 300 µg of peptides per sample, we identified 22,712 phosphorylation sites, of which 19,075 were localized with high confidence and 1,203 are phosphorylated tyrosine residues, representing 6.3% of all detected phospho-sites. HiRIEF fractions with the most acidic isoelectric points are enriched in multiply phosphorylated peptides, which represent 18% of all the phospho-peptides detected in the pH range 2.5–3.7. Cross-referencing with the PhosphoSitePlus database reveals 1,264 phosphorylation sites that have not been previously reported and kinase association analysis suggests that a subset of these may be functional during the mitotic phase.

## Introduction

Protein phosphorylation is a fundamental regulatory mechanism in eukaryotic cells, affecting various processes such as cell growth, proliferation, survival, migration and metabolism. Signals governing these processes can be transduced through cascades of protein phosphorylation and dephosphorylation, enzymatically governed by protein kinases and protein phosphatases. Deregulated protein phosphorylation is involved in the development of many diseases, including inherited disorders caused by mutated protein kinases^1^ and neurodegenerative diseases such as Alzheimer’s or Parkinson’s disease where hyper-phosphorylation causes proteins to misfold and aggregate^2^. Importantly, protein phosphorylation is critically deregulated in cancer, where protein kinase activity uncoupled from extra-cellular growth stimulatory signals results in constitutive firing of growth pathways and uncontrolled proliferation^3^. Our understanding of the intracellular phosphorylation circuits can thus increase our knowledge of both physiological and pathological cellular processes and can be applied to develop new therapeutic strategies.

Profiling of phosphorylation changes on a large scale (phosphoproteomics) can be performed by mass-spectrometry (MS). However, identification of phosphorylated peptides is challenging due to their low abundance and poor ionization efficiency^4^, thus methodological improvements are needed to increase the analytical depth. Current methods include reduction of sample complexity both by extensive sample fractionation and by specific phospho-peptide enrichment strategies^5^. These approaches most often demand long MS-analysis time and high amount of starting material, hence influencing throughput and cost of the analysis, and may not even be feasible in cases where the availability of starting material is limited. Labeling strategies allow parallel quantitative analysis of multiple samples (multiplexing), therefore reducing the analysis time as compared with sequential sample analysis performed with label-free approaches. Furthermore, multiplexing also results in reduced quantitative variability^6, 7^. Metabolic labeling methods, especially SILAC, have been broadly employed for phosphoproteomics analysis^5^, but their multiplexing capability is limited to two or three samples. Isobaric labels, such as Tandem Mass Tags (TMT), allow simultaneous quantification of up to ten samples. A limited number of phosphoproteomics studies utilizing isobaric labeling have been reported so far, identifying in between 11,000 to 26,000 phosphorylation sites^8–13^, however they require high amount of starting material or labeling reagents.

Previously, we described a method for precise fractionation of peptides based on isoelectric point (high-resolution isoelectric focusing, HiRIEF). Furthermore, we demonstrated that when coupled to liquid chromatography and mass spectrometry (HiRIEF LC-MS), the method enables high coverage of the cellular proteome^14^. In the current study, we explore how HiRIEF LC-MS performs in a quantitative phosphoproteomics workflow. Samples from HeLa cells were enriched for phosphorylated peptides employing titanium dioxide (TiO_2_), followed by isobaric labeling and HiRIEF LC-MS. Performance of the developed workflow resulted in the identification of 22,712 phosphorylation sites across 10 samples, of which 1,264 were not previously reported in the PhosphoSitePlus database^15^. Finally, functionality of such novel phosphorylation sites was predicted to indicate sites with putative biological functions.

## Results

### HiRIEF LC-MS allows for in depth identification and accurate quantification of cellular protein phosphorylation events

**Figure 1:**
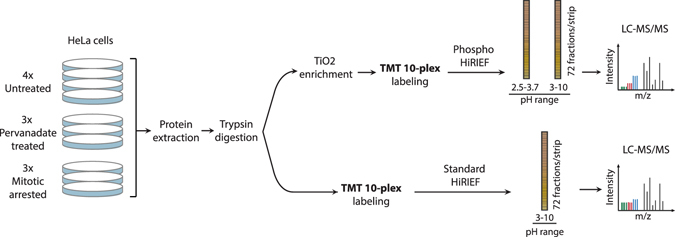
Experimental workflow applied to perform HiRIEF-based phosphoproteomics analysis. Protein extracts from HeLa cells untreated (4 replicates), pervanadate treated or arrested in mitosis (3 replicates each) were digested to peptides with trypsin. For Phospho HiRIEF analysis, samples were enriched for phosphorylated peptides with titanium dioxide (TiO_2_), labeled with Tandem Mass Tags (TMT) and pooled. The pooled sample was split in two and fractionated by HiRIEF using immobilized pH gradient (IPG) strips with pH range 2.5–3.7 (ultra-acidic strip) and 3–10 (wide-range), prior to LC-MS analysis. For standard proteomics analysis, peptide samples were labeled with TMT, pooled, fractionated by HiRIEF using a wide-range (3–10) IPG strip and analyzed by LC-MS.

The HiRIEF pre-fractionation method enables in-depth proteome analysis by highly reducing the peptide complexity in the fractions analyzed by LC-MS^14^. In order to investigate how this method performs in a quantitative phosphoproteomics setting, we combined it with titanium dioxide based phospho-peptide enrichment and isobaric labeling (Fig. 1). Isobaric labeling of peptides using TMT 10-plex reagents allows for relative quantification of up to ten different samples in a single MS experiment. Control cells were harvested in exponential growth phase (four biological replicates) to represent the phosphoproteome in an asynchronous cell population (Supplementary Fig. S1b). Experimental conditions included pervanadate treatment and mitotic arrest (three biological replicates each) and were selected to enrich for different phosphorylation events. Pervanadate inhibits protein tyrosine phosphatases^16, 17^, resulting in dramatically increased levels of tyrosine phosphorylation as assayed by western blot (Supplementary Fig. S1a), without affecting the cell cycle distribution (Supplementary Fig. S1b). Mitotic arrest achieved by double thymidine block and nocodazole treatment induces extensive serine and threonine phosphorylation^18^. Flow cytometric cell cycle analysis confirmed the efficiency of the induction of mitotic arrest (Supplementary Fig. S1b). The human cervix adenocarcinoma cell line HeLa was used as a model system as it is a well characterized model for phosphorylation studies, especially in relation to mitosis^18–20^.

Since measured phosphorylation changes may be influenced by alteration of total protein abundances, standard proteomics analysis was included in the experimental workflow. Peptides were prepared from the ten samples using a slightly modified filter-aided sample preparation (FASP) protocol. For standard proteomics analysis, an aliquot from each of the ten peptide samples was labeled using TMT 10-plex isobaric labels, pooled and fractionated by HiRIEF using a wide-range (pH range 3–10) immobilized pH-gradient (IPG) strip prior to analysis with LC-MS (Fig. 1, Standard HiRIEF LC-MS). For phosphoproteomics analysis, phospho-peptides were enriched from the individual ten peptide samples using titanium dioxide beads, labeled using TMT 10-plex, pooled and fractionated by HiRIEF using ultra-acidic (pH range 2.5–3.7) or wide-range IPG strips prior to analysis with LC-MS (Phospho HiRIEF LC-MS). All the IPG strips were divided into 72 fractions and extracted to 96-well plates, with the fraction numbering proceeding from the acidic end to the basic end of the strip. The total analysis time was approximately 89 hours for the standard proteomics and 163 hours for the phosphoproteomics analysis, and the total peptide amounts used per sample were 50 μg and 300 μg respectively (Table 1).

**Table 1:** Analysis conditions and number of identifications for Phospho HiRIEF LC-MS and Standard HiRIEF LC-MS.

	Phospho HiRIEF LC-MS			Standard HiRIEF LC-MS
	Ultra-acidic (IPG 2.5–3.7)	Wide-range (IPG 3–10)	Combined analysis	Wide-range (IPG 3–10)
Peptide amount per sample (μg)	150	150	300	50
No. of analyzed fractions	72	60	132	72
Total analysis time (h)	88.8	74	162.8	88.8
No. of unique peptides*	8,200	26,336	32,015^‡^	82,705
No. of quantified unique peptides*	7,338	25,234	30,177^‡^	82,606
No. of unique phospho-peptides*	7,338	16,153	21,377^‡^	—^§^
No. of quantified unique phospho-peptides*	6,537	15,294	19,815^‡^	—^§^
No. of quantified unique proteins*^†^	3,091^†^	4,735^†^	5,224^†‡^	9,350
No. of quantified unique genes*^†^	3,003^†^	4,548^†^	5,116^†‡^	9,182
No. of peptides/protein (gene-centric)	2.18	3.37	3.87	9.00

The column “Combined analysis” recapitulates the overall results of the Phospho HiRIEF LC-MS approach, obtained by employing both immobilized pH gradient (IPG) strips, pH range 2.5–3.7 and 3–10.

*Peptides are defined as unique by sequence and number of phosphorylations; proteins and genes are defined as unique by Uniprot-ID and Gene symbol, respectively.

^†^For Phospho HiRIEF LC-MS analysis the reported numbers refer only to proteins found to carry phosphorylated amino acids, and their corresponding genes.

^‡^Number of unique identifications obtained across both IPG strips.

^§^Phosphorylation was not included as dynamic modification when searching data generated by the Standard LC-MS analysis.

**Figure 2:**
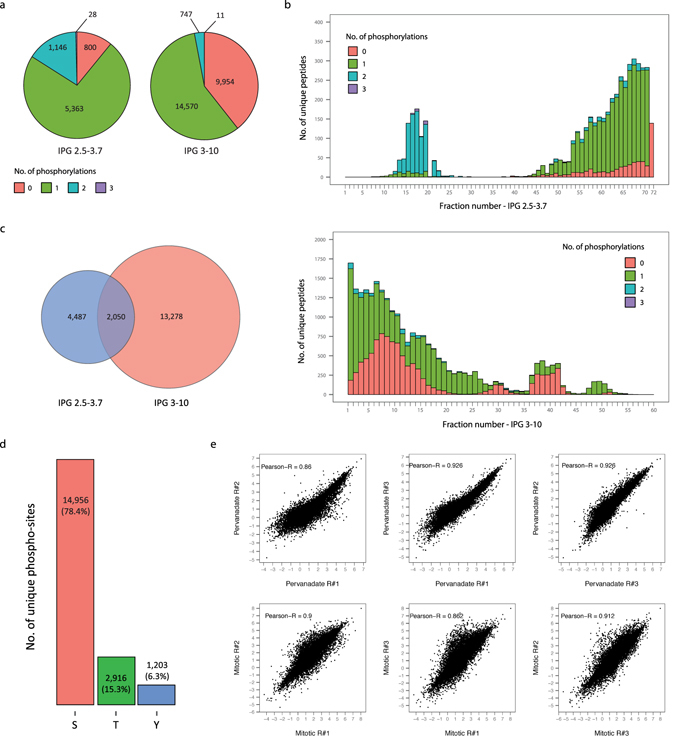
Identifications by and reproducibility of HiRIEF based phosphoproteomics. (**a**) Pie chart representing the number of unique peptides broken down by number of phosphorylations. (**b**) Distribution of unique peptides across HiRIEF fractions, broken down by number of phosphorylations. Fraction numbering proceeds from the acidic end towards the basic end of the strips. (**c**) Venn diagram showing the overlap between unique phospho-peptides identified with the two HiRIEF pH ranges, 2.5–3.7 and 3–10. (**d**) Total number of identified unique phospho-sites, displayed by modified residue (serine, threonine or tyrosine). (**e**) Correlation of phospho-sites log_2_ transformed ratio values for each replicate pair; Pearson correlation coefficient is displayed. Ratio values were calculated using as a denominator the average of the four untreated samples, and normalized to total protein levels (see Methods).

Phospho HiRIEF LC-MS analysis of the peptides separated with the ultra-acidic and with the wide-range IPG strip led to the identification of 7,338 and 16,153 unique phospho-peptides respectively. Of those, 6,537 and 15,294 phospho-peptides are quantified in all 10 TMT channels and are employed for subsequent analyses. Additionally, 18% and 5% of the phospho-peptides identified from the ultra-acidic and from the wide-range IPG strip respectively are multiply phosphorylated (Fig. 2a). Examination of the number of identifications per fraction across the analyzed pH ranges demonstrates uneven peptides distribution, due to the naturally biased isoelectric point distribution of tryptic peptides (Fig. 2b). Peptides carrying two or more phosphorylations are identified predominantly from the acidic end (first 30 fractions) of the ultra-acidic IPG strip, whereas the remaining fractions of the ultra-acidic IPG strip as well as the IPG 3–10 strip contained mainly singly phosphorylated peptides. The higher proportion of multiply phosphorylated peptides found in the ultra-acidic IPG strip results from decreases in peptide isoelectric point due to the additional phospho-groups. To further characterize such decreases in isoelectric point determined by phosphorylation, we examined the fraction shift induced by single or double phosphorylation on peptides identified in the wide-range IPG strip. Non phosphorylated counterparts of 5,125 singly phosphorylated and 94 doubly phosphorylated peptides were identified by Standard HiRIEF LC-MS analysis. The majority of these peptides can be classified into four groups according to the degree of acidity/basicity of their amino acidic composition (number of D, E, K, H and R residues). Each group of peptides displays a characteristic fraction shift determined by phosphorylation; the shift varies between 8 and 55 fractions and is smaller for acidic peptides and greater for basic peptides. Additionally, double phosphorylation induces a larger decrease in isoelectric point than single phosphorylation; upon double phosphorylation peptides shift 6 to 9 fractions more than upon single phosphorylation (Supplementary Fig. S2).

Finally, a subset of 2,050 phospho-peptides commonly identified in both IPG strips (Fig. 2c) is attributed to the overlap in their pH ranges (Supplementary Fig. S3), demonstrating non-redundant fractionation of peptides based on their isoelectric point.

Across both experiments 22,712 unique phosphorylation sites corresponding to 5,111 genes were identified, of which 19,075 sites were localized with high confidence (pRS score > = 95). Furthermore, 1,203 unique tyrosine phosphorylation sites were identified, corresponding to 6.3% of the total number of identified sites (Fig. 2d), similar to what previously reported using a pTyr affinity capture approach^20^. The Standard HiRIEF LC-MS experiment resulted in the identification and quantification of 9,350 proteins corresponding to 9,182 genes, identified with a protein level FDR of 2.6% (Supplementary Data S1). For both Phospho HiRIEF LC-MS and Standard HiRIEF LC-MS, quantifications for each TMT channel are expressed as ratios relative to the average of the f`our untreated samples. Upon pervanadate treatment protein ratios distribute tightly around zero, indicating that very little regulation occurs at the total protein level. Samples from mitotic arrested cells, in contrast, present a wider ratio distribution corresponding to differences in protein abundance between the untreated (asynchronous cell population) and the mitotic cells (Supplementary Fig. S4). To account for total protein level changes, phospho-site ratios are normalized to their corresponding total protein ratios (see Methods section). Out of 19,075 phosphorylation sites localized with high confidence, 18,382 sites correspond to 4,566 genes that are also identified by Standard HiRIEF LC-MS analysis. Normalized phospho-site ratios are calculated for this subset of 18,382 sites and are highly reproducible (Pearson correlation coefficients for every pair of replicates is near 0.9) (Fig. 2e and Supplementary Data S2).

### HiRIEF phosphoproteomics enlarges the catalogue of human phosphorylation sites and is proficient at identifying lowly abundant modifications

**Figure 3:**
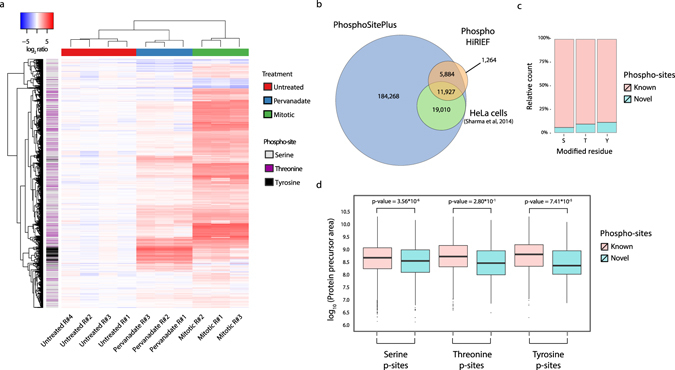
Functional characterization of the identified phospho-sites. (**a**) Heatmap representing complete linkage hierarchical clustering based on Euclidian distance of the unique phospho-site ratios. Row color-coding represents the modified residue (S,T or Y). (**b**) Venn diagram showing the overlap between the identified phospho-sites, the ones previously reported in the PhosphoSitePlus database and the HeLa phospho-sites reported by Sharma et al. in 2014. (**c**) Proportion of novel and known phospho-sites per modified residue. (**d**) Distribution of protein precursor areas for the novel and known phospho-sites per type of modified residue. Two-sided t-test was performed to assess significance.

Hierarchical clustering based on complete linkage and Euclidian distance separates the samples based on treatment and reveals a tight cluster of the up-regulated phospho-tyrosine sites in pervanadate treated cells (Fig. 3a). However, separate analysis of phosphorylated tyrosine sites highlights increased TMT signal in two of the mitotic-arrested samples compared to the third replicate of the same condition (TMT channels 130 N and 130 C compared to 131) (Supplementary Fig. S5a). According to vendor information (Thermo Fisher Scientific, Rockford, IL, Supplementary Data S3), TMT channels 130 N and 130 C are affected by isotopic impurities contained in the TMT reagents 129 N and 129 C respectively, both of which correspond to pervanadate treated samples. The effect of TMT reagent isotopic impurities is usually negligible (proportion of reagent impurities ~1–5%), but becomes significant in this case where tyrosine sites in pervanadate treated samples have TMT intensities an order of magnitude higher than in the other experimental conditions. Mitotic arrested cells display a characteristic high signal in serine and threonine phosphorylation, consistent with previous reports^18^, with a typical low impact on the quantification of the other samples (Supplementary Fig. S5b).

Cross-referencing the sites identified in this analysis with those contained in the human subset of the PhosphoSitePlus database^15^ reveals 1,264 sites that have not been previously reported (6.6% of all the identified sites). Moreover, comparison with HeLa cells specific phosphorylation sites included in the database from a recent in-depth HeLa phosphoproteomics study^20^ shows an overall overlap of 62% (Fig. 3b). The discovered novel phospho-sites included 132 phospho-tyrosine sites corresponding to 126 genes (Supplementary Data S4). Among those, we found tyrosine phospho-sites contained within protein functional domains of receptor-type tyrosine-protein phosphatase eta protein (PTPRJ), tyrosine-protein phosphatase non-receptor type 12 (PTPN12) and intersectin-1 (ITSN1). In particular, phosphorylation of Y48 and Y1058 in PTPN12 and PTPRJ respectively, located in the protein tyrosine phosphatases catalytic domains, possibly affect the proteins’ enzymatic activities. Similarly, phosphorylation of Y1012 in a Src Homology 3 (SH3) domain of ITSN1 may modulate its interaction with other proteins. Furthermore, identified novel phospho-sites are skewed towards tyrosine residues as 5.5%, 9% and 11% of serine, threonine and tyrosine sites respectively are novel (Fig. 3c). Finally, HiRIEF phosphoproteomics is adept at locating phosphorylations on low abundance proteins as the median precursor area of proteins carrying novel serine and tyrosine phosphorylation sites is significantly lower than that of proteins carrying previously described sites (Fig. 3d). This result is in contrast with a previous report indicating tyrosine phosphorylation to be biased towards high abundance proteins^20^, suggesting that technical improvements (such as pre-fractionation methods with higher resolution or novel enrichment strategies) allows for identification of low abundance phospho-tyrosine sites.

### Integration of kinase-substrate prediction and systems biology analyses suggest novel phosphorylation sites with a putative functionality in the cell cycle regulatory network

To define phospho-sites significantly regulated upon pervanadate treatment or mitotic arrest we used a Benjamini-Hochberg corrected t-test p-value of less than 0.01 and a fold change of more than two when comparing treated to control cells. After pervanadate treatment and cell cycle arrest 3,254 and 11,031 phospho-sites were found significantly regulated respectively (Supplementary Fig. S6). To examine whether phosphorylation sites are differentially regulated when identified in singly or in multiply phosphorylated peptides, we examined the subset of 1,810 phospho-sites that were commonly identified in peptides carrying single or multiple phosphorylations. Upon mitotic arrest, 130 of those phospho-sites are significantly regulated when identified in singly phosphorylated peptides but are not regulated when identified in multiply phosphorylated peptides. Conversely, 613 phospho-sites are significantly regulated only when identified in multiply phosphorylated peptides but not in singly phosphorylated peptides (Supplementary Fig. S7). Such regulation events that are detected exclusively in the context of multisite phosphorylation would not be apparent when analyzing only singly phosphorylated peptides, thus illustrating the value of detecting multiply phosphorylated peptides.

**Figure 4:**
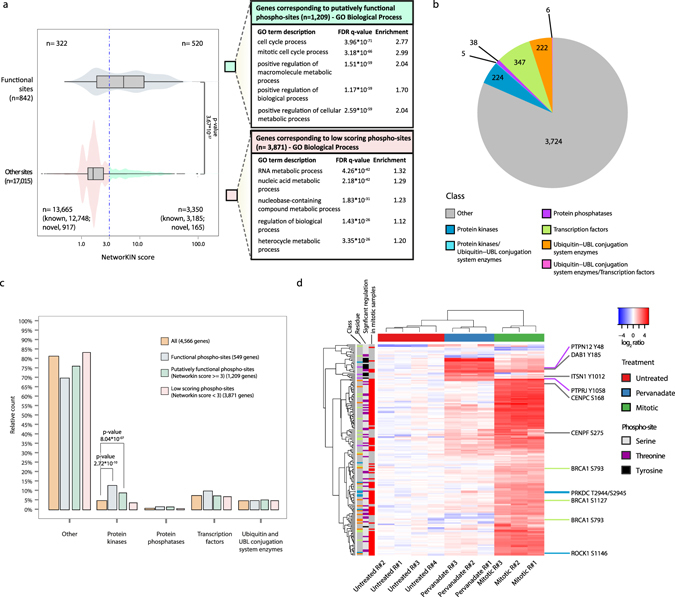
Novel phospho-sites have putative roles in mitotic regulation. (**a**) NetworKIN score distributions for the subsets of known functional phospho-sites or phospho-sites with no known function (Other sites). Two-sided t-test was performed to assess significance. A threshold score of 3 is used to separate a population of putatively functional phospho-sites and GO Biological Process enrichment analysis is performed on the score separated populations. (**b**) Number of genes identified for each of the examined classes in the phospho-HiRIEF analysis. Genes ascribable to more than one class are indicated in separate groups, with the classes separated by a “/” character. (**c**) Relative distribution of genes across classes for the subsets of genes corresponding to functional, putatively functional or low-scoring phospho-sites. Class enrichment in each subset compared to the class distribution for all genes identified in the Phospho-HiRIEF analysis is evaluated by Fisher exact test. Genes attributable to more than one class were excluded from this analysis. (**d**) Heatmap representing hierarchical clustering based on Euclidian distance and complete linkage of putatively functional novel phospho-sites. Row color-codings represent: “Class”, class of the protein carrying the indicated phospho-site (color coding as in Fig. 4b); “Residue”, modified residue; “Significant regulation in mitotic samples”, red – significant, grey - not significant. Detailed list of the phospho-sites included in the heatmap is provided in Supplementary Data S5.

The function of the majority of the reported phosphorylation sites is not presently known and different approaches have been undertaken to predict site functionality. Evolutionary conservation, participation in protein-protein interactions, regulation of adjacent post-translational modifications or mediation of protein domains’ activity have been shown to be valuable features to screen for functional phospho-sites^21–23^. Recently, kinase association strength was hypothesized to also contribute in predicting site functionality^21, 24^. Machine learning models integrating kinase association strength, evolutionary conservation and structural information were employed to predict phospho-site functionality and the relative contribution of each feature was evaluated. The strength of association to kinases, measured by NetworKIN score, was shown to contribute the most in determining the models’ predictions^24^. The NetworKIN score represents the likelihood of a phospho-site being modified by a certain kinase^25^, and is constructed by combining kinase-substrate interaction information from the STRING database^26^ and consensus sequence motif information from the NetPhorest linear phospho-motif atlas^27^. As NetworKIN score was reported to distinguish functional phospho-sites^24^, we tested this principle in our dataset. Kinase association was predicted for the subset of identified phosphorylation sites with previously reported function (“functional”), as defined in PhosphositePlus (842 phospho-sites), and for the remaining identified sites (17,015 phospho-sites). The distribution of NetworKIN scores for the functional phospho-sites have a significantly higher mean than the distribution of scores for the other sites (Fig. 4a, left panel). Therefore, NetworKIN score was employed to predict phospho-site functionality; among the group of 17,015 phosphorylation sites without a previously reported function, phospho-sites with a NetworKIN score of 3 or more were defined as “putatively functional”. Genes whose protein product carried those putatively functional phospho-sites are enriched in the Gene Ontology (GO) terms “Cell cycle process” and “Mitotic cell cycle process” as compared to all the identified genes. Genes corresponding to phospho-sites with NetworKIN scores <3 (“low-scoring phospho-sites”) instead show enrichment in “RNA metabolic process”, but with lower fold (Fig. 4a, right panel). Additionally, putatively functional phosphorylation sites corresponding to genes annotated with the GO term “Cell cycle process” are more often significantly regulated upon mitosis arrest than putatively functional sites corresponding to other genes (Supplementary Fig. S8).

Genes corresponding to the phospho-sites identified in the phospho-HIRIEF analysis encode for proteins falling into several classes based on information retrieved from publicly available databases. Classes considered were protein kinases, protein phosphatases, transcription factors and ubiquitin and ubiquitin-like (UBL)-conjugating system enzymes, containing respectively 224, 38, 347 and 222 genes identified in the Phospho HiRIEF LC-MS analysis (Fig. 4b). To further evaluate the results from the NetworKIN analysis, we applied Fisher’s exact test to assess enrichment in any of these classes for the genes corresponding to functional, putatively functional or low-scoring phospho-sites, using all genes identified in the phospho-HIRIEF analysis as reference set (background). We found that the list of genes corresponding to phosphorylation sites with previously reported function (functional sites) is enriched in the class of protein kinases as compared to the reference set. Interestingly, also the subset of genes corresponding to putatively functional phospho-sites is enriched in protein kinases, supporting their hypothesized functionality (Fig. 4c). Hierarchical clustering was re-applied on the subset of novel putatively functional phosphorylation sites (165 unique phospho-sites) to examine their regulation, showing that many of these sites are significantly regulated upon mitotic arrest (Fig. 4d). These sites were also annotated according to the protein functional domain, if any, where the phosphorylated residue is localized, resulting in a list of sites of possible biological relevance (Supplementary Data S5 and indicated in Fig. 4d).

**Figure 5:**
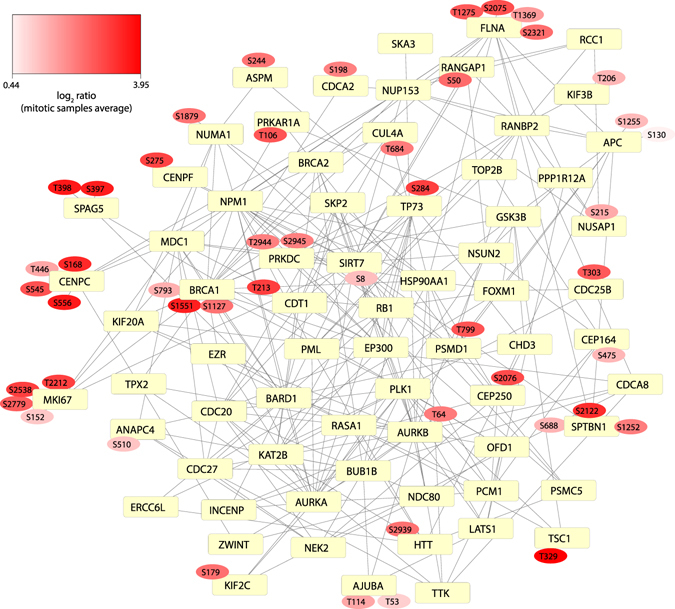
Cell cycle related novel putatively functional phospho-sites are highly connected in a protein-protein interaction network. The network represents interactions between proteins containing novel putatively functional phospho-sites annotated with the GO term “Cell cycle process”. Interactions of these proteins with other “Cell cycle process” annotated proteins are also included (see Methods for description of the network generation process). Square shaped nodes symbolize proteins (annotated by gene symbol) while round shaped nodes symbolize phosphorylation sites (detailed list available in Supplementary Data S5). Phosphorylation sites are color-coded by the average log_2_ normalized ratio value across the three mitotic arrested samples. The network was generated using the Cytoscape software platform and the PhosphoPath plugin^28^. Protein interactions are retrieved by PhosphoPath based on the BioGRID database^66^.

Finally, to examine the possible role of the novel putatively functional phospho-sites in cell cycle regulation, a protein-protein interaction network was generated using the Cytoscape application PhosphoPath^28^. The network includes genes corresponding to novel putatively functional phospho-sites and annotated with the GO term “Cell cycle process” and other closely connected genes with the same GO annotation (see Methods for details on the network generation). As illustrated in Fig. 5, genes involved in the cell cycle process which display novel putatively functional phosphorylation sites have a high degree of connectivity, suggesting that many regulatory mechanisms within this process may still be unknown. Moreover, several genes in this network display novel putatively functional phosphorylation sites localized on protein functional domains, including members of the kinetochore plate CENPC and CENPF, the protein kinase PRKDC and the ubiquitin ligase BRCA1 (Supplementary Data S5).

## Discussion

In this study we applied isobaric labeling and HiRIEF fractionation to perform quantitative phosphoproteomics and standard proteomics analysis, identifying 19,075 phosphorylation sites with high confidence localization, of which 1,203 are phospho-tyrosine sites. Genes encoding for proteins identified in the phosphoproteomics and standard proteomics analysis were overlapped to normalize phosphorylation sites to total protein quantities, resulting in 18,382 quantified phospho-sites. Multiplex quantification by TMT allows for comparison of up to ten experimental conditions in a single run, therefore reducing analysis time and peptide amount required per sample. Previous efforts have quantified 11,000 to 17,000 phospho-sites by applying phospho-enrichment on 5–10 mg of peptides, followed by TMT labeling^9–12^. Labeling prior to phospho-enrichment resulting in 13,000–26,000 quantified phospho-sites has also been reported, but it is costly as it required 2–15 sets of labeling reagents^8, 13^.

In the current work, 300 μg of peptides per sample were enriched for phosphorylated peptides by TiO_2_ prior to labeling, which required a single TMT set. Enrichment prior to labeling might introduce quantitative variability, however it highly diminishes required amounts of TMT reagents and therefore the cost of the analysis. Peptides from each sample were enriched by TiO_2_ in batch, reducing sample handling as compared with the majority of reported phosphoproteomics workflows where chromatographic fractionation is applied before enrichment^5^. Importantly, the aforementioned benefits of the Phospho HiRIEF workflow enable phosphoproteomics analysis of samples where metabolic labeling is difficult and sample amount is limited, such as phosphoproteomics analysis of clinical materials.

Chromatographic pre-fractionation methods commonly employed for phosphoproteomics analysis are Strong Cation Exchange (SCX) and more recently high-pH reversed phase chromatography^29, 30^. Isoelectric point (pI) based peptide pre-fractionation in combination with phosphoproteomics analysis has so far only been investigated in a very limited number of studies. Hung *et al*. fractionated mixtures of three methyl-esterified synthetic peptides in different phosphorylation statuses on strips with pH range 3–8, demonstrating that phosphorylated peptides can be separated from unmodified peptides based on isoelectric point^31^. In another work, peptides derived from whole cell lysates were focused on an IPG strip ranging from pH 3 to 6, identifying 58 phospho-peptides overall^32^. Here we used a broad pH range (pH 2.5–3.7 and pH 3–10) separated into narrow pH fractions to analyze the distribution of phospho-peptides demonstrating at a high resolution a distinct fractionation pattern according to the number of phosphorylations (Supplementary Fig. S2). Multiply phosphorylated peptides are prevalently localized in the acidic end (first 30 fractions) of the ultra-acidic strip, therefore reducing ionization competition by singly phosphorylated peptides, which focus on the remaining pH range regions up to fraction 55 of the IPG 3–10 strip. Additionally, we observe a considerable proportion of phosphorylation sites that are significantly regulated only when identified in multiply phosphorylated peptides (Supplementary Fig. S7), demonstrating that the identification of those peptides contributes additional information on regulated events upon treatment. Multisite phosphorylation is involved in regulating several cellular processes including protein degradation and signaling cascades^33–39^, and is implicated in neurodegenerative disorders^40, 41^. Isoelectric focusing based fractionation represents therefore a useful strategy in such settings to study coordinated regulation of phosphorylation at multiple sites in close vicinity. Finally, phospho-peptide identifications common between the two strips are mostly derived from regions of overlapping pH ranges (Supplementary Fig. S3). In summary, both ionization competition and sample complexity are reduced by applying HiRIEF fractionation, making it a valuable addition to current approaches for detection of phosphorylated peptides.

Altogether we identified 14,956 serine, 2,916 threonine and 1,203 tyrosine phosphorylation sites, of which 1,264 are novel. Novel serine and tyrosine sites are located on proteins of lower abundance than the ones containing known phospho-sites. This suggests that previous methods have been biased towards the detection of phospho-sites on highly abundant proteins, while identification of lowly abundant phospho-sites necessitates methodological developments, such as improved fractionation or enrichment strategies. In particular, incomplete coverage of phospho-tyrosine sites by previous methods is also indicated by the higher proportion of novel phosphorylation sites in the subset of tyrosine compared to serine and threonine phospho-sites. The potential of methodological improvements for the study of tyrosine phosphorylation is exemplified by the recent identification of >10,000 pTyr sites across 9 cell lines, of which 36% were novel, with a new tyrosine enrichment method based on a SH2-domain-derived pTyr superbinder^42^.

Finally, we used the generated data to investigate phosphorylation events specific to the mitotic phase. During the mitotic phase anabolic functions such as mRNA transcription, processing and translation are inactivated^43, 44^. Inhibition of proteins responsible for these processes has been suggested to be mediated by extensive protein phosphorylation^18^, potentially by impairment of inter- and intra-molecular interactions due to the multiple negative charges added onto the proteins. These inhibitory phosphorylation events are thought to be mediated by low affinity interactions with kinases as opposed to functional phosphorylation events that need to be regulated quickly and precisely by high affinity interactions with kinases^21, 24, 45^. In agreement with this theory, we show that the subset of phosphorylation sites that are predicted to interact poorly with kinases (“low scoring”) occur mainly on proteins involved with mRNA processing. In contrast, a subset of phospho-sites with high affinity for kinases (“putatively functional”) occurs on proteins related to cell cycle progression.

Lack of knowledge on phospho-sites functionality is a major issue in moving from omics data to targeted molecular studies. For this reason, we overlaid systems biology analysis with manual curation of the data to provide a list of phosphorylation sites of possible biological relevance. Novel putatively functional phospho-sites were annotated with respect to the protein class and to the protein domain on which they occur. Phosphorylation of particular protein domains such as binding or catalytic domains is often biologically relevant, as it can alter protein interactions and enzymatic activity. A well known example is regulation of protein kinase activity by phosphorylation of the activation loop within the kinase domain or of the region flanking it^46^. Another example is phosphorylation of binding and adaptor domains such as SH3 domains (or of any binding interface), impacting protein-protein interactions^47, 48^. Moreover, we analyzed the regulation of novel putatively functional phospho-sites during mitosis and their role in the context of the cell cycle regulatory network. We believe that the provided list of novel putatively functional phosphorylation sites is of interest for future studies on regulation of phospho-signaling during mitotic progression.

In conclusion, the method presented here enables phosphoproteomics analysis compatible with multiplexed isobaric labeling, and provides for good analytical depth and sensitivity compared with previous studies while requiring lower amounts of starting material. We therefore believe it can contribute to further revealing the function and impact of protein phosphorylation on cellular signaling and disease processes.

## Methods

### Cell Culture and Treatment

HeLa cells (ATCC CCL-2) were cultured in RPMI-1640 AQmedia supplemented with 10% (v/v) fetal bovine serum, penicillin and streptomycin to a final concentration of 100 IU/ml and 100 μg/ml respectively (all from Sigma-Aldrich). Tyrosine phosphatase activity was inhibited by 15 minute treatment with 1 mM pervanadate (Sigma-Aldrich), as described previously^49^.

A mitotic arrested population was obtained by applying a double thymidine block to synchronize cells in the G1 phase, followed by releasing the block and treating with 0.1 μg/ml nocodazole overnight upon commencement of mitosis^19, 50^.

### Cell Cycle Analysis

The efficiency of mitotic arrest was monitored by fluorescence-activated cell sorting (FACS) analysis. Cells were detached with trypsin-EDTA solution (0.5 g/l porcine trypsin, 0.2 g/l EDTA) and fixed by a minimum of 1 h incubation in 70% ethanol at 4 °C. Cells were centrifuged at 180 *g*, the pellet resuspended in phosphate buffered saline (PBS), pelleted again, and suspended in buffer containing 0.1% (v/v) Triton X-100 to permeabilize the plasma membranes. Following treatment with RNase (0.1 mg/ml), cells were treated with propidium iodide (PI, 100 μg/ml) to stain DNA. Samples were analyzed on a BD FACSCalibur cytometer (Becton Dickinson, San Jose, CA), using the FL2 filter (588/40 nm) to measure PI. About 10,000 events were acquired per sample and cell aggregates (doublets) were excluded based on the FL2 signal width, as described before^51^.

### Sample Preparation

Cellular proteins were extracted when cells were near 70% confluence. Culture dishes were rinsed twice with ice-cold PBS and cells were lysed by scraping in presence of 0.5% sodium deoxycholate (SDC) and 0.35% sodium lauroyl sarcosinate (SLS) with Halt Protease and Phosphatase Inhibitor Cocktail (Thermo Fisher). Lysates were sonicated and centrifuged for 15 min at 14,000 *g* and 4 °C. Supernatants were transferred to new vials and protein levels quantified using the DC-protein assay (Bio-Rad Laboratories, Hercules, CA, USA).

### Western Blot Analysis

Western blots were performed to evaluate the extent of tyrosine phosphorylation upon pervanadate treatment. Proteins were separated on NuPAGE Bis-Tris gels using the Novex gel electrophoresis system (Invitrogen, Life Technologies) and transferred onto Amersham Hybond C Extra nitrocellulose membranes (GE Healthcare, Waukesha, WI, USA). Membranes were then blocked with 5% milk (Bio-Rad Laboratories), incubated with primary antibody overnight at 4 °C, washed in 0.1% Tween-20/Tris-buffered saline, and incubated with horseradish peroxidase (HRP)-conjugated secondary antibody (anti-mouse, NA931V, or anti-rabbit, NA934V) from GE Healthcare for 1 h at room temperature. After an additional wash, membranes were incubated with a chemiluminescent detection reagent (Amersham ECL prime reagent), and imaged using ImageQuant LAS 4000 (GE Healthcare). The following primary antibodies were used: p-Tyr pY20 (sc-508) and actin I–19 (sc-1616) from Santa Cruz Biotechnology (Santa Cruz, CA, USA).

### Protein digestion

The protein extracts were processed following the filter-aided sample preparation (FASP) protocol^52^ with slight modifications. Briefly, cysteine residues were reduced with 1 mM dithiothreitol and alkylated with 5.5 mM iodoacetamide in solution. Subsequently, protein extracts were applied on 10 k filtration units (Merck-Millipore), centrifuged and washed once before digestion at 37 °C overnight with trypsin/Lys-C mix 1:25 w/w (Promega, Madison, WI, USA). Wash and digestion steps were performed using a 0.2% SDC-50 mM HEPES solution. The peptides were collected by centrifugation of the FASP filters and the SDC was precipitated by addition of 0.5% trifluoroacetic acid (TFA). Finally, the samples were desalted using Polymeric Reversed Phase-Solid Phase Extraction (RP-SPE) cartridges (Phenomenex) and peptide concentration was measured with DC protein assay (BioRad). Separate 300 μg and 50 μg aliquots of peptides per sample were lyophilized and set aside for phospho-peptide enrichment, and Tandem Mass Tag (TMT) labeling for total protein analysis respectively.

### Phospho-peptide enrichment

Phospho-peptide enrichment was performed using STAGE tips loaded with TiO_2_ beads (GL Sciences, Tokyo, Japan), as previously reported^53^. Beads were prepared as a slurry (50 μg/μl) in binding buffer (80% acetonitrile, ACN, 6%, TFA). Peptides were dissolved in 200 μl of binding buffer, bead slurry was added to a 1:4 ratio of peptides to TiO_2_ beads, and the mixture was incubated while rotating for 30 minutes at room temperature. The beads were then loaded on STAGE tips and the unbound fraction was collected by centrifuging for 1 minute at 100 *g*. The beads were then washed once with binding buffer and twice with wash buffer (80% ACN, 0.1% TFA). Phospho-peptides were eluted with 2 × 100 μl of 5% ammonia buffer into vials containing 200 μl of 10% formic acid (FA) to neutralize the pH. Eluates were lyophilized and desalted using RP-SPE cartridges (Phenomenex).

### Tandem Mass Tag labeling

Peptide samples were dissolved in 100 mM triethylammonium bicarbonate buffer (TEAB) at pH 8.5. 10-plex Tandem Mass Tag (TMT) (Thermo Fisher Scientific, Rockford, IL) reagents were dissolved in 40 μl ACN, added to each sample, and incubated for 3 hours at room temperature with gentle shaking. The efficiency of labeling was determined by liquid chromatography tandem mass spectrometry (LC-MS) prior to pooling samples.

Pooled samples were desalted with RP-SPE cartridges (Phenomenex) and then lyophilized in a speedvac prior to focusing on immobilized pH gradient (IPG) gel strips (GE Healthcare Bio-Sciences AB, Uppsala, Sweden).

### Peptide level high-resolution isoelectric focusing

High-resolution isoelectric focusing (HiRIEF) was performed as described previously^14^ using linear pH ranges of 2.5–3.7 (“ultra-acidic” range, a strip prototype provided by GE Healthcare Bio-Sciences AB, Uppsala, Sweden) or 3–10 (“wide-range”, commercially available from GE Healthcare). The buffer capacity of both IPG 2.5–3.7 and IPG 3–10 strips is >3 mEq/L per pH unit, which ensures high loading capacity and high resolution of separated peptides. The sample containing TMT-labeled phospho-peptides was split in two and pre-fractionated using the ultra-acidic and wide-range IPG strips (Phospho HiRIEF). The sample not enriched for phosphorylated peptides was fractionated using the wide-range IPG strip (Standard HiRIEF). Strips were divided into 72 fractions (fraction numbering proceeds from the strip acidic end to the basic end) and extracted to V-bottom 96-well plates with a liquid handling robot (GE Healthcare prototype modified from Gilson liquid handler 215). Plates were lyophilized in a speedvac prior to LC-MS analysis.

### Liquid chromatography tandem mass spectrometry

To examine phospho-peptides using HiRIEF (Phospho HiRIEF LC-MS), all 72 fractions from the ultra-acidic IPG strip plate along with the first 60 fractions from the wide-range IPG strip plate were analyzed with LC-MS (no phospho-peptides were identified in fractions 61 to 72 in optimization experiments, data not shown).

For Standard HiRIEF, all 72 fractions from the wide-range IPG strip plate were analyzed with LC-MS (Standard HiRIEF LC-MS). Each HiRIEF fraction was dissolved in 15 µl of phase A (95% water, 5% dimethylsulfoxide (DMSO), 0.1% FA), mixed by drawing/dispensing 10 µl ten times, followed by the auto sampler (Ultimate 3000 RSLC system, Thermo Scientific Dionex) injecting 10 µl into a C18 guard desalting column (Acclaim pepmap 100, 75 µm × 2 cm, nanoViper, Thermo). Following 5 min of flow at 5 µl/min driven by the loading pump, the 10-port valve switched to analysis mode in which the binary high-pressure gradient pump (referred to as nano gradient, NG, pump) provided a flow of 250 nL/min through the guard desalting column. From an initial composition of 3% phase B (90% ACN, 5% DMSO, 5% water, 0.1% formic acid) the reversed phase gradient proceeded to 45% phase B over 50 min. Upon completion of the gradient, the column was washed with a solution of 99% phase B for 10 min and re-equilibrated to the initial composition. Total LC-MS run time was 74 min. A nano EASY-Spray column (pepmap RSLC, C18, 2 µm bead size, 100 Å, 75 µm internal diameter, 50 cm long, Thermo Scientific) was used on the nano electrospray ionization (NSI) EASY-Spray source (Thermo Scientific) at 60 °C. Online LC-MS was performed using a hybrid Q-Exactive mass spectrometer (Thermo Scientific). Fourier transform-based mass spectrometer (FTMS) master scans with a resolution of 70,000 (and mass range 300–1,700 m/z) were followed by data-dependent MS/MS (35,000 resolution) on the 5 most abundant ions using higher energy collision dissociation (HCD) at 30% normalized collision energy. Precursor ions were isolated with a 2 m/z window. Automatic gain control (AGC) targets were 1 * 10^6^ for MS1 and 1 * 10^5^ for MS2. Maximum injection times were 100 ms for MS1 and 150 ms (for proteomics) or 400 ms (for phosphoproteomics) for MS2. The entire duty cycle lasted ~1.5 s. Automated precursor ion dynamic exclusion was used with a 60 s duration. Precursor ions with unassigned charge states or a charge state of +1 were excluded. An underfill ratio of 1% was applied.

### Proteomics database search

All tandem mass spectrometry (MS/MS) spectra were searched by Sequest/Percolator under the Proteome Discoverer software platform (PD 1.4, Thermo Scientific) using a target-decoy strategy. The reference database was the human protein subset of Swissprot, release 2017-02-06, 42,147 entries. Precursor ion and product ion mass tolerances of 10 ppm and 0.02 Da respectively were used for HCD-FTMS. Additionally, peptide spectral matches (PSMs) allowed for up to two missed trypsin cleavages (Lys-Pro and Arg-Pro were not considered cleavage sites). Carbamidomethylation on cysteine and TMT 10-plex on lysine and N-terminus were set as fixed modifications and oxidation of methionine was set as a dynamic modification while searching all MS/MS spectra. Phosphorylation of serine, threonine and tyrosine were included as dynamic modifications while searching MS/MS spectra from the Phospho HiRIEF LC-MS analysis. Quantitation of TMT 10-plex reporter ions was performed using an integration window tolerance of 10 ppm. A false discovery rate cutoff of 1% was applied at the peptide level. The phosphoRS algorithm node was added to the workflow for the phosphoproteomics search to obtain probabilities of localization of phosphorylation sites^54^, and only sites with high confidence of localization (> = 95 pRS score) were used for quantification. The mass spectrometry proteomics data have been deposited to the ProteomeXchange Consortium via the PRIDE partner repository with the dataset identifier PXD005410.

### Data analysis

For both Phospho HiRIEF LC-MS and Standard HiRIEF LC-MS, only peptide spectral matches (PSMs) with a complete set of TMT reporter ions are employed for ratios calculation. Ratios were calculated first for all PSMs by dividing the intensity of each TMT channel by the average intensity of the four TMT channels corresponding to the untreated samples. Phospho-site ratios for Phospho HiRIEF LC-MS quantitative analysis are represented as the median of all PSM ratios for a unique phospho-site. Phospho-sites are displayed as 15 amino acid sequences centered at the phosphorylated residue (sequence window). Analogously, protein ratios for Standard HiRIEF LC-MS quantitative analysis are represented as the median over all PSM ratios for a unique gene. Furthermore, protein ratios are normalized to the median of all proteins per TMT channel, assuming equal protein loading of all 10 samples. Phospho-site levels are normalized to changes in protein abundance by subtracting the log_2_ transformed protein ratio from the log_2_ transformed phospho-site ratio. For peptide count and plotting of the distribution across HiRIEF fractions, peptides were defined as unique by amino acidic sequence and number of phosphate groups.

For Standard HiRIEF LC-MS, protein precursor areas are expressed as the average of the top three peptide precursor areas for each gene. Peptides were assigned to the HiRIEF fraction where they were identified with the highest precursor area. Protein level FDR was estimated using the “picked” protein FDR approach, as described previously^55^. Information on TMT reagents isotopic impurities was obtained from the vendor (Thermo Fisher Scientific, Rockford, IL, Lot number: PB199188D) and is available in Supplementary Data S3.

### Bioinformatics analysis

Plots were generated using RStudio. Lists of human phosphorylation sites and of functional phosphorylation sites were obtained from the PhosphoSitePlus database, release 2017-02-16^15^. A list of 30,937 HeLa cells phosphorylation sites identified by Sharma *et al*.^20^ with high confidence of localization (localization score > = 95) was obtained from the Curated Information of the PhosphoSitePlus database.

Kinase association was predicted for the identified phospho-sites using NetworKIN^25^ for 17,857 out of 18,382 high-confident and quantified phosphorylation sites. Kinase association could not be predicted for some phosphorylation sites because the corresponding proteins have no reported interaction in STRING or their Uniprot identifier couldn’t be mapped. For each phospho-site, the prediction with the highest NetworKIN score was used to represent the strength of kinase association^24^. NetworKIN scores are normalized using the distribution of scores across the whole proteome for each kinase, so that a score of 3 for a particular phosphorylation site-kinase interaction represent a prediction that is better than 99.99% of all the predictions for that kinase in the whole proteome^56^. A Networkin score of 3 or more can therefore be employed to separate confident predictions^57–59^. Gene ontology (GO) enrichment analysis was performed with the web service GOrilla by selecting the “Two unranked lists of genes” option with the list of genes identified by the Standard HiRIEF approach as background set and setting a p-value threshold of 10^−3^. Genes were assigned to different classes using publicly available databases for protein kinases^60^, protein phosphatases^61^, transcription factors^62–64^ and enzymes belonging to the ubiquitin and ubiquitin-like (UBL) conjugation systems^65^. Enrichment analysis for these classes was performed with Fisher’s exact test to calculate p-values.

Network analysis of the phosphorylated proteins was performed using the Cytoscape software platform with the PhosphoPath plugin^28^. Briefly, a “.phos” data file generated according to the instructions specified in https://github.com/linseyr/PhosphoPath was imported to Cytoscape. The data file includes 304 genes corresponding to the identified putatively functional phospho-sites and annotated with the GO term “Cell cycle process” (as in Fig. 4a, top-right panel) and their corresponding phosphorylation sites. Kinase-substrate and protein-protein interactions information were obtained using the PhosphoPath database import option and are based on PhosphoSitePlus^15^ and BioGRID database^66^ (all interactions were considered) respectively. Based on these data, a network visualization was created and a sub-network containing the 32 genes corresponding to proteins carrying novel putatively functional phospho-sites was extracted from it, in which 22 genes are directly connected to each other. Nine of the remaining genes and some of their first neighbors from the original network were included by manual curation to achieve a more comprehensive representation of the connectivity between all the cell cycle related genes corresponding to novel putatively functional phosphorylation sites.

## Electronic supplementary material

Supplementary PDF File

Dataset 1

Dataset 2

Dataset 3

Dataset 4

Dataset 5
