# Supplementary material for: Isoelectric point-based fractionation by HiRIEF coupled to LC-MS allows for in-depth quantitative analysis of the phosphoproteome
Source: Sci Rep. 2017 Jul 3;7:4513. doi: 10.1038/s41598-017-04798-z (PMC5495806; doi:10.1038/s41598-017-04798-z)
Supplement: Supplementary file 1 — Supplementary PDF File [file 41598_2017_4798_MOESM1_ESM.pdf]

Supplementary Material

Isoelectric point-based fractionation by HiRIEF coupled to LC-MS allows for in depth quantitative analysis of the phosphoproteome

Elena Panizza[1], Rui MM Branca[1], Peter Oliviusson[2], Lukas M Orre[1], and Janne Lehtiö[1]*

[1] Department of Oncology-Pathology, Science for Life Laboratory, Karolinska Institutet, Stockholm, Sweden
[2] GE Healthcare Bio-Sciences AB, Uppsala, Sweden

*To whom correspondence should be addressed:
Janne Lehtiö, PhD, Professor, Karolinska Institutet, Department of Oncology-Pathology,
Science for Life Laboratory, 171 65 Stockholm, Sweden
e-mail: janne.lehtio@ki.se

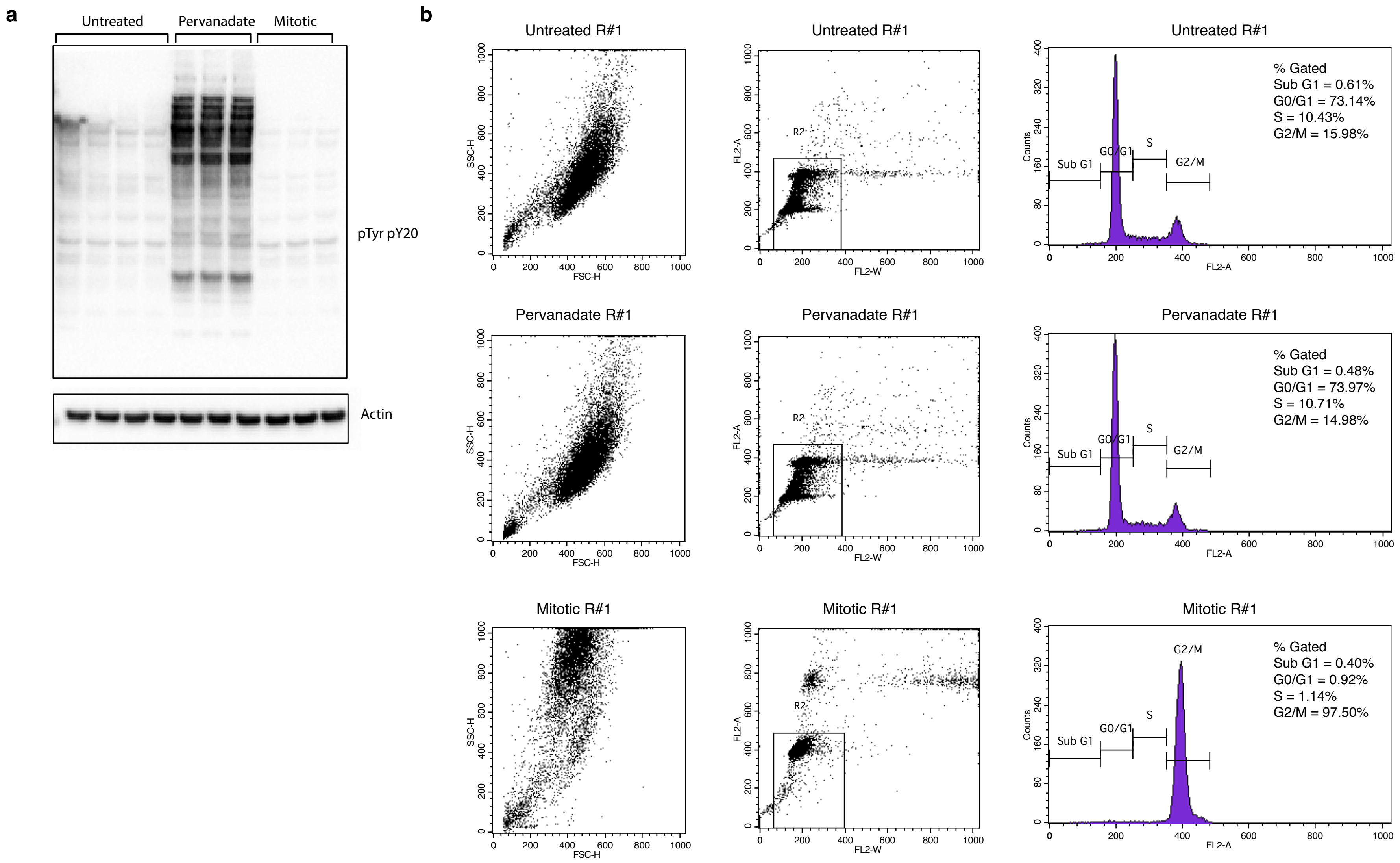

Supplementary Figure S1. Validation of the cell treatments. (a) Western blot analysis using an antibody recognizing phosphorylated tyrosine residue (PY20), shows a very high signal in the pervanadate treated samples compared to the untreated or the mitotic arrested samples. Actin protein abundance was examined to verify the consistency of the total protein loading across samples. **(b)** FACS analysis was performed to visualize the fluorescent signal derived from propidium iodide (PI). The area of the signal measured using the FL2 filter (FL2-A) reflects the content of DNA of the cell and is used to assign events to different cell cycle phases. Cell aggregates were excluded based on the FL2 signal width (FL2-W), by setting a gate in the FL2-A vs. FL2-W scatter plot on events corresponding to single cells (see Methods). The figure illustrates one replicate per experimental condition, as an example. More than 96.8% of the cells that underwent double thymidine block and nocodazole treatment have double DNA content in each of the three replicates.

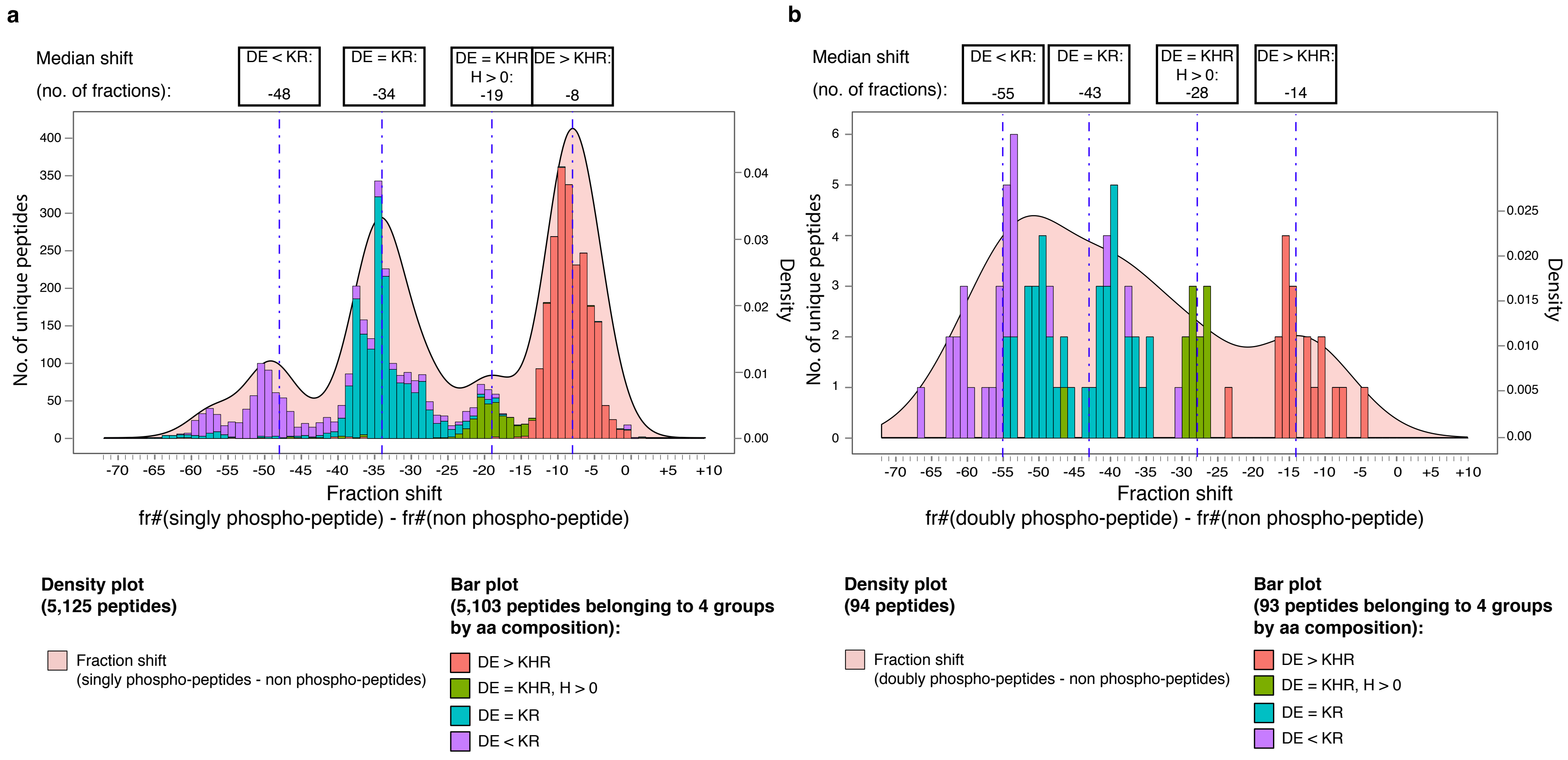

Supplementary Figure S2. Decreases in isoelectric point determined by single or double phosphorylation. Decrease in isoelectric point is described as fraction shift (fr#(phosphorylated peptide) - fr#(non phosphorylated peptide)). Peptides are grouped according the degree of acidity/basicity of their sequences. Each group displays characteristic median fraction shifts induced by the addition of one and two phospho-groups. **(a)** Fraction shift of 5,125 singly phosphorylated peptides identified in the IPG 3-10 by Phospho HiRIEF LC-MS for which the corresponding non phosphorylated peptides were identified by Standard HiRIEF LC-MS. **(b)** Fraction shift of 94 doubly phosphorylated peptides identified in the IPG 3-10 by Phospho HiRIEF LC-MS for which the corresponding non phosphorylated peptides were identified by Standard HiRIEF LC-MS.

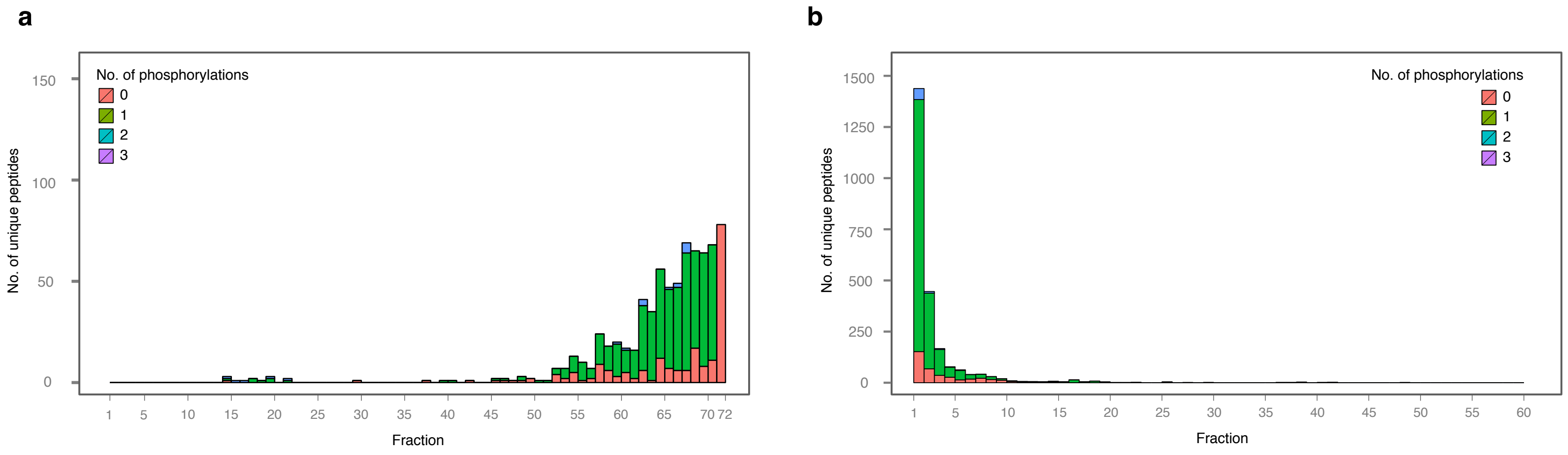

Supplementary Figure S3. Distribution across HiRIEF fractions of unique peptides overlapping between the two strips. Distribution of unique peptides across HiRIEF fractions, broken down by number of phosphorylations. Fraction numbering proceeds from the acidic end towards the basic end of the strips. **(a)** HiRIEF strip pH range 2.5-3.7. **(b)** HiRIEF strip pH range 3-10.

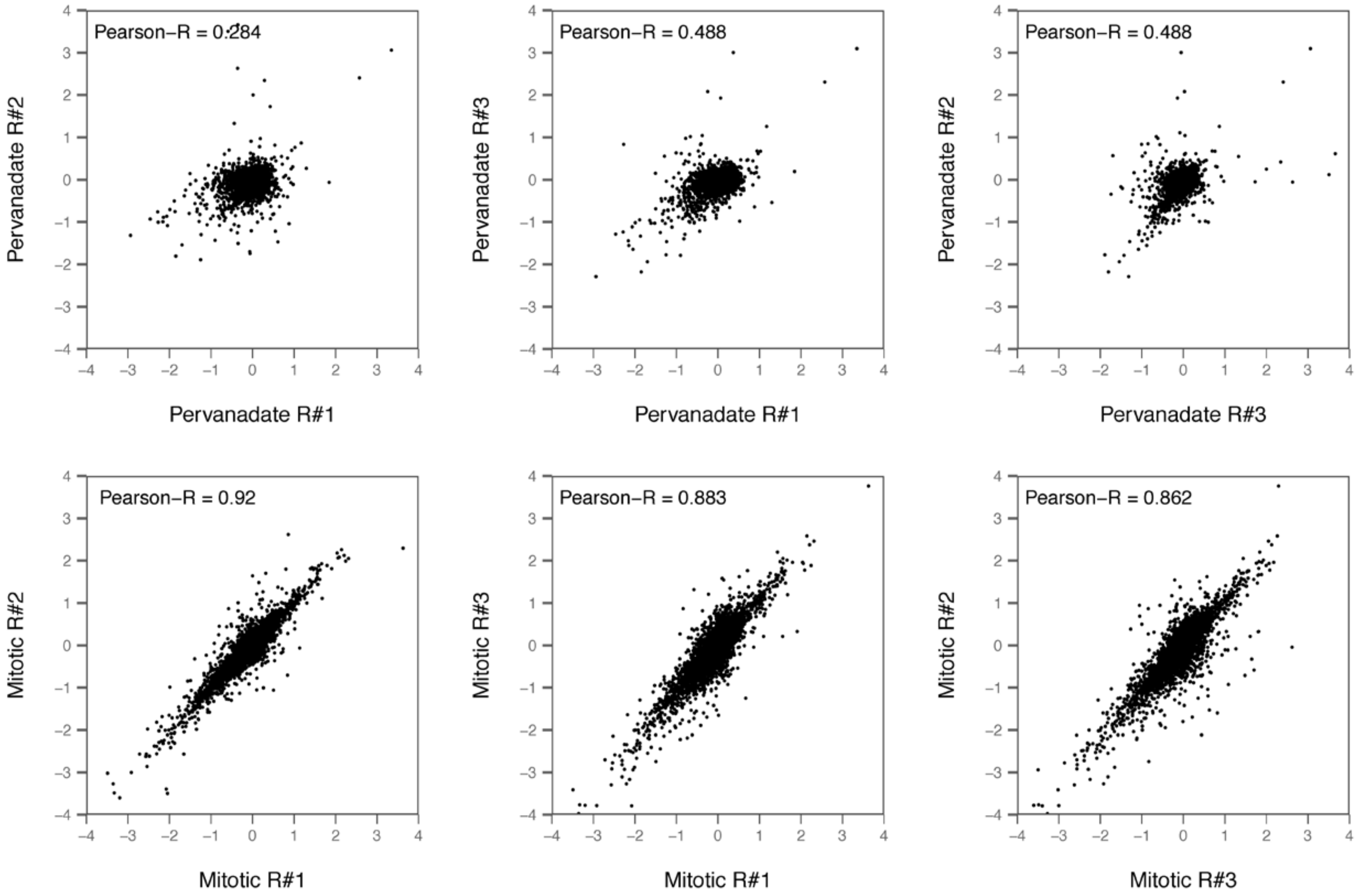

Supplementary Figure S4. Standard HiRIEF protein ratios distribution. Scatter plots representing gene-centric protein log2 transformed ratio values for each replicate pair with their Pearson correlation coefficient displayed. Ratio values were calculated using as a denominator the average of the four untreated samples (see Methods).

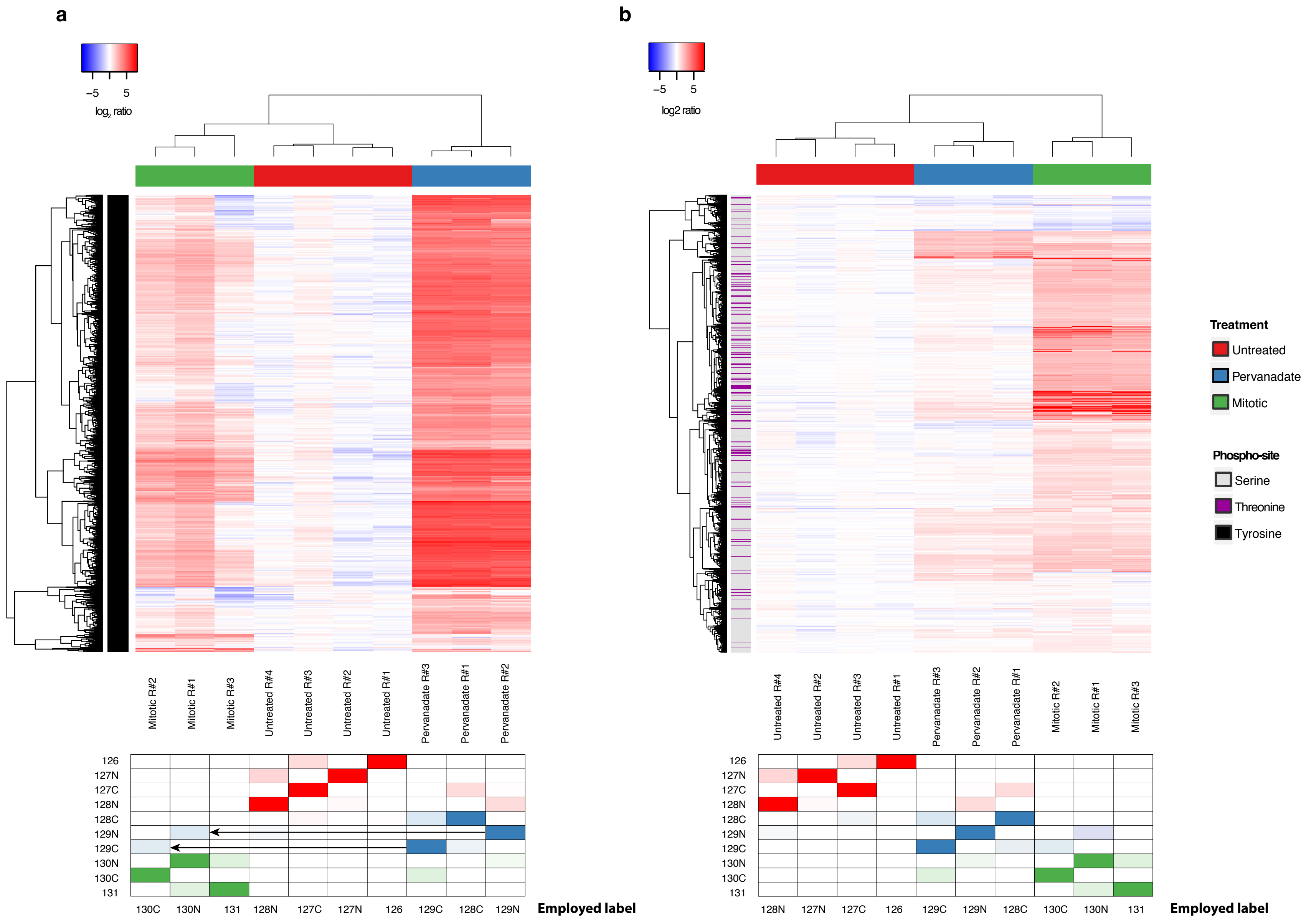

Supplementary Figure S5. Tyrosine and serine/threonine phospho-site ratios clustering analysis. (a) Heatmap representing complete-linkage hierarchical clustering based on Euclidian distances of unique tyrosine phospho-sites. Bottom panel represents a map of TMT reporter ion isotopic distributions (-2, -1, 0, +1, +2) for carbon isotopes, as provided by the vendor (Lot Number: PB199188D). Color shading is proportional to the reporter ion intensity in each TMT channel per each employed TMT label. **(b)** Heatmap representing complete-linkage hierarchical clustering based on Euclidian distances of unique serine and threonine phospho-sites. Row color-coding represents the modified residue (S or T). Bottom panel as in **(a)**.

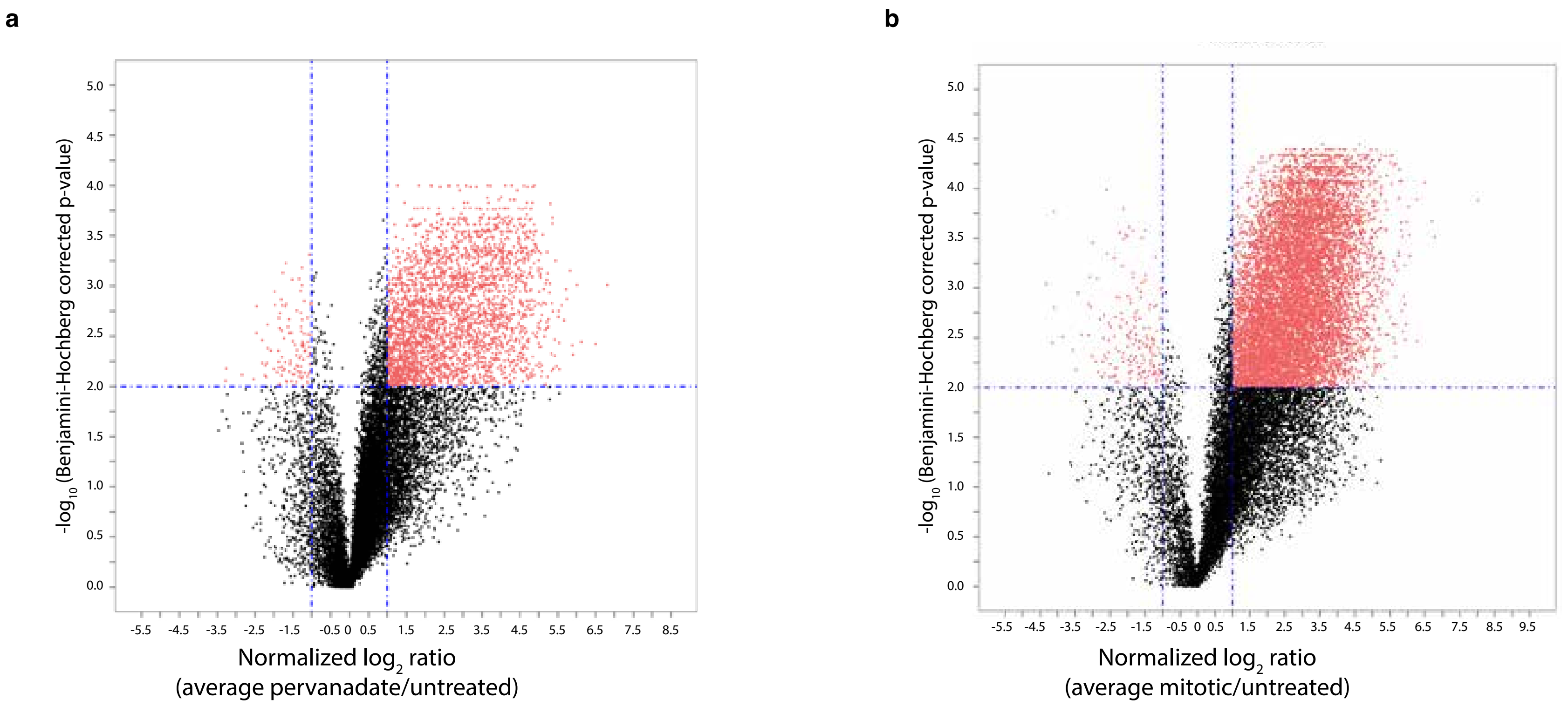

Supplementary Figure S6. Selection of significantly regulated phospho-sites quantified by the Phospho HiRIEF LC-MS analysis. Volcano plots representing the selection of significantly regulated events for each experimental condition. The average log2 transformed ratio of the three replicates is plotted for each experimental condition. Benjamini-Hochberg corrected p-values lower than 0.01 and 2 fold change up- or down-regulations were deemed significant. **(a)** Phospho HiRIEF, pervanadate treated vs. untreated samples. **(b)** Phospho HiRIEF, mitotic arrested vs. untreated samples.

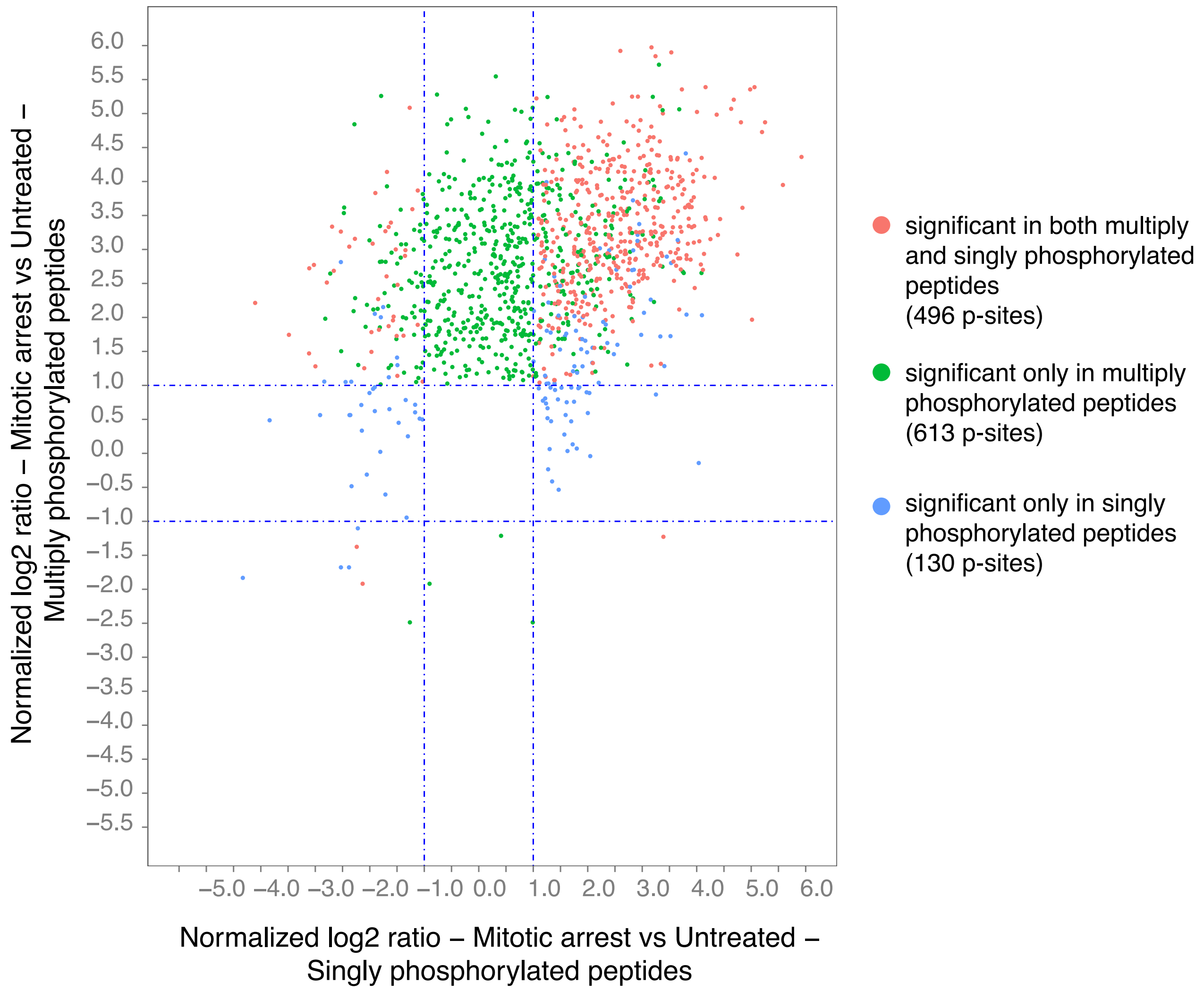

Supplementary Figure S7. Significantly regulated phospho–sites identified in singly and multiply phosphorylated peptides. Correlation between log_2 ratios of phospho-sites significantly regulated upon mitotic arrest identified in singly and multiply phosphorylated peptides (1,239 phospho-sites). 49.9% of the phospho-sites are significantly regulated when identified in multiply regulated phosphorylated peptides but not in singly phosphorylated peptides (green coloured dots).

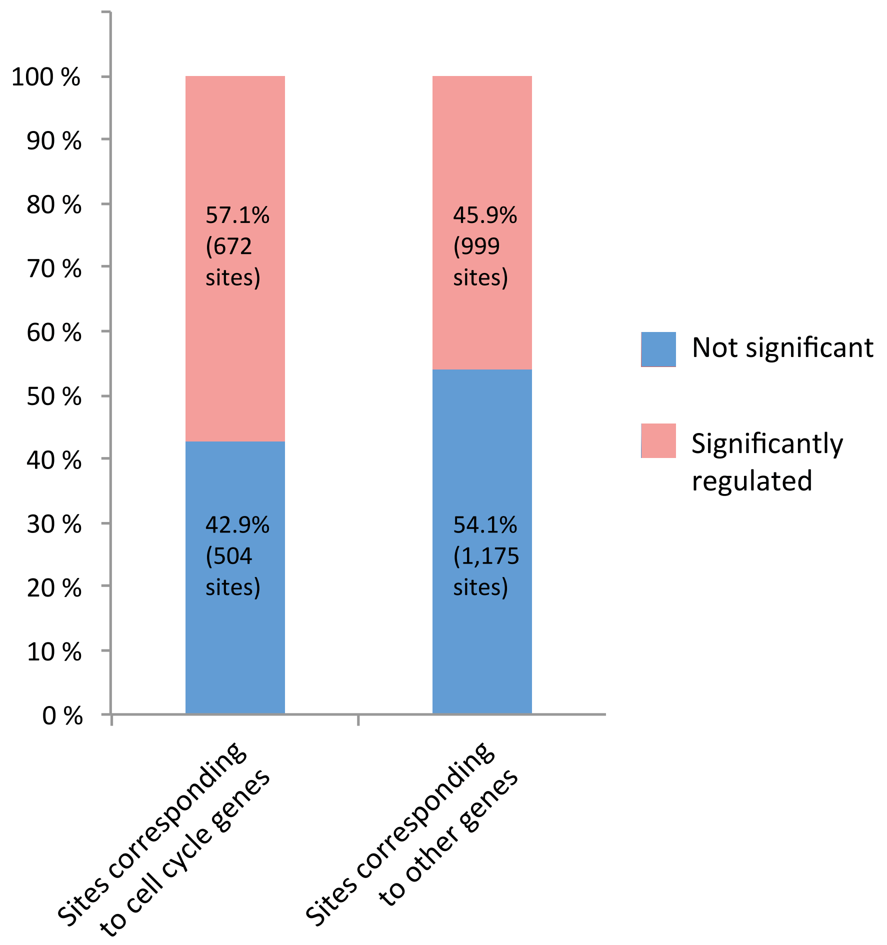

Supplementary Figure S8. Regulation of putatively functional phosphorylation sites upon mitotic arrest. In the subset of putatively functional sites, 57.1% of the phospho-sites corresponding to genes annotated with the GO term “cell cycle process” (cell cycle genes) are significantly regulated upon mitotic arrest as compared to only 45.9% of sites corresponding to other genes.
